# Mechanical milling: a sustainable route to induce structural transformations in MoS_2_ for applications in the treatment of contaminated water

**DOI:** 10.1038/s41598-018-37798-8

**Published:** 2019-01-30

**Authors:** Maria Cantarella, Giuliana Gorrasi, Alessandro Di Mauro, Mario Scuderi, Giuseppe Nicotra, Roberto Fiorenza, Salvatore Scirè, Maria Elena Scalisi, Maria Violetta Brundo, Vittorio Privitera, Giuliana Impellizzeri

**Affiliations:** 1 CNR-IMM, Via S. Sofia 64, 95123 Catania, Italy; 2 Department of Industrial Engineering, University of Salerno, Via Giovani Paolo II 132, 84084 Fisciano, Salerno Italy; 3 CNR-IMM, Z.I. VIII Strada 5, 95121 Catania, Italy; 4 Department of Chemical Sciences, University of Catania, Viale Andrea Doria 6, 95125 Catania, Italy; 5 Department of Biological, Geological and Environmental Sciences, University of Catania, Via Androne 81, 95124 Catania, Italy

## Abstract

Two-dimensional (2D) nanomaterials have received much attention in recent years, because of their unusual properties associated with their ultra-thin thickness and 2D morphology. Besides graphene, a new 2D material, molybdenum disulfide (MoS_2_), has attracted immense interest in various applications. On the other hand, ball-milling process provides an original strategy to modify materials at the nanometer scale. This methodology represents a smart solution for the fabrication of MoS_2_ nanopowders extremely-efficient in adsorbing water contaminants in aqueous solution. This work reports a comprehensive morphological, structural, and physicochemical investigation of MoS_2_ nanopowders treated with dry ball-milling. The adsorption performances of the produced nanopowders were tested using methylene blue (MB) dye and phenol in aqueous solution. The adsorption capacity as a function of ball-milling time was deeply studied and explained. Importantly, the ball-milled MoS_2_ nanopowders can be easily and efficiently regenerated without compromising their adsorption capacity, so to be reusable for dye adsorption. The eventual toxic effects of the prepared materials on microcrustacean *Artemia salina* were also studied. The present results demonstrate that ball-milling of MoS_2_ offers a valid method for large-scale production of extremely efficient adsorbent for the decontamination of wastewaters from several pollutants.

## Introduction

Stimulated by the numerous studies devoted to exploit the extraordinary properties of graphene, recently, other two-dimensional (2D) materials are attracting a lot of attention. Among the 2D materials, emerging transition metal dichalcogenides and in particular, molybdenum disulfide (MoS_2_), have received enormous interest because of its unique structural, thermal, mechanical and electronic properties^1^. MoS_2_ possesses a layered structure consisting of layers of S-Mo-S, where a Mo atom layer is sandwiched by two layers of S atoms. Crystals of MoS_2_ are composed of vertically stacked layers held together by relatively weak van der Waals interactions in a hexagonally-packed structure. MoS_2_ is a semiconductor with an indirect band-gap of ~1.2 eV in multilayer structure that turns into a direct band-gap of ~1.9 eV upon processing to a single layer^2^. Furthermore, MoS_2_ has a good chemical stability and remains stable in acids, alkalis and organic solvents^3–5^. Thanks to its properties MoS_2_ in a few years imposed itself as an attractive material for several applications, such as for electronic transistors, lubrication, catalysis, energy storage, sensors, photodetectors, etc^1,6^. Recently, many studies are also directed to the use of MoS_2_ nanostructures for water treatment applications^7–9^.

Water purification is still one of the most serious problem in the world. Indeed, augmented agricultural and industrial activity has produced an increased contamination of our limited water resources due to the dispersion of various organic pollutants, such as industrial dyes, aromatic compounds, and heavy metal ions^10^. Consequently, numerous efforts are emerged to develop cheap and efficient technologies for water treatment, able to guarantee abundant clean water and create environmental and public health sustainability^11–18^. Currently, adsorption of pollutants through activated carbon is one of the most popular method for water treatment due to its effectiveness. However, commercial activated carbon is relative expensive due to the high production cost, high regeneration cost that requires high-pressure stream, and 10–15% loss in the reactivation procedure^19,20^.

Nanostructured MoS_2_ can be a valid alternative to the conventional materials for water treatment^21^. Some researchers have focused their attention to the use of MoS_2_ nanosheets as photocatalyst for the degradation of organic pollutants and bacteria inactivation^7,22,23^. As mentioned before, single layers of MoS_2_ are characterized by band-gap values that make MoS_2_ a potential visible-light photocatalyst. Charge carriers are generated in the valence and conduction bands under visible light irradiation. However, the recombination rate of the photo-generated electron-hole pairs is extremely high making smaller the photocatalytic efficiency. To reduce this phenomenon, the realization of nanocomposites with large band-gap metal oxide in which MoS_2_ is used as co-catalyst is inevitable to really degrade the contaminants through a photocatalytic mechanism^24–28^. Another interesting feature of MoS_2_ is the capacity to act as a very efficient adsorbent for organic molecules. The source of these adsorption abilities is due to the stacked planar structure of MoS_2_. The adjacent planes of MoS_2_, held together by van der Waals interactions, allow molecules or atoms to infiltrate and diffuse freely between the layers where they adsorb^29^. This extraordinary property of MoS_2_ has not been exploited extensively, despite the adsorption process through nanostructured materials is considered an efficient and economical method for the removal of contaminants from water at large-scale^30,31^. To date, only few examples are present in the literature in which MoS_2_ is efficiently used as adsorbent for water treatment, and they involve the realization of sophisticated nanostructures or the functionalization of MoS_2_ surface in order to increase the number of adsorption sites^32–46^.

In this work, we treated commercial MoS_2_ nanopowders with a dry ball-milling process. We demonstrated that this simple and highly-scalable method^47^ can significantly enhance the adsorption properties of the MoS_2_ without the need of any additional reaction or involvement of chemical agents. Some examples of ball-milling process applied to MoS_2_ to exfoliate bulk MoS_2_^48–50^ or to modify the electrochemical and catalytic properties^51–53^ have been already reported in the literature. However, to our knowledge, there are not studies focused on the application of ball-milling to produce efficient MoS_2_ adsorbents. The ball-milled materials were deeply characterized by scanning electron microscopy (SEM), N_2_ adsorption-desorption measurements, transmission electron microscopy (TEM), X-ray diffraction (XRD) analyses, and X-ray photoelectron spectroscopy (XPS). The adsorption capacity was tested using methylene blue (MB) dye and phenol as representative water pollutants. In addition, potential toxic effects of the MoS_2_ nanopowders on microcrustacean *Artemia salina* were originally evaluated.

## Methods

### Materials

Commercial molybdenum disulfide nanopowders (MoS_2_), with a theoretical diameter of 90 nm (99% trace metal basis) were purchased from Sigma-Aldrich and hereafter simply called “as received”.

Methylene Blue (MB, 0.05 wt% in H_2_O) and phenol (C_6_H_5_OH, ≥99.5%) were also purchased from Sigma-Aldrich.

### Ball-Milling procedure

As received MoS_2_ powders (3 g) were milled in a planetary ball milling PM100 (Retsch) at ambient temperature. The jar volume was 50 cm^3^, the used balls (5) were of tungsten carbide, the milling rate equal to 450 rpm and the milling time up to 40 h. The machine was interrupted every 4 hours and the material mixed up with a tongue.

### Characterization methods

SEM analyses were performed with a field emission Zeiss Supra 25 microscope operating at 5 kV.

The N_2_ adsorption-desorption experiments were carried out in a Sorptomatic 1990 Micropore configuration (Thermo Quest). Before tests, the samples were degassed at 120 °C at 10^−3^ Torr. The physisorption experiments were performed at liquid nitrogen boiling temperature (77 K). Surface area and pore size distribution were evaluated with the help of a fully computerized unit attached to the Micropore unit system, using the Brunauer-Emmett-Teller (BET) equation and the Dollimore-Heal method, respecitvely.

The nanoscale morphological and structural characterization of MoS_2_ nanopowders was carried out by TEM. The analyses were performed with a Cs-probe-corrected JEOL JEM ARM200CF microscope at primary beam energy of 200 keV operated in scanning TEM (S/TEM) mode. As for the specimen preparation, the powders were dispersed in isopropyl alcohol by sonication for 15 min, thereafter 5 µL of each dispersion were deposited on an Au TEM grid with a lacey carbon support film. In order to obtain images of MoS_2_ nanopowders with Z-contrast, the high-angle annular dark field mode (HAADF) was used with a convergence semi-angle of 33 mrad and a collection semi-angle in a range comprised between 64 mrad and 172 mrad.

XRD patterns of MoS_2_ nanoparticles were detected by a Bruker D-500 diffractometer at 40 kV and 40 mA, using a Cu Kα line of 0.15418 nm wavelength. The angle of incidence was fixed at 2.5°, and the diffractogram was collected from 10 to 60°. The XRD patterns were analyzed by the Bruker software suite, including ICSD structure database.

XPS measurements were performed by a PHI ESCA/SAM 5600 Multy technique spectrometer with the use of a Mg standard X-ray source. The pressure in the chamber was ~10^−9^ Torr. The analyses were carried out at 45° photoelectron takeoff angle relative to the sample surface with an acceptance angle of ±3°. The analyzer pass energy was set at 23.5 eV for the high resolution of all the spectra in the regions of C 1 s, O 1 s, Mo 3d and S 2p. The binding energy (BE) scale was calibrated by centering the C 1 s signal of the aliphatic/aromatic component at 285.0 eV.

### Adsorption test

The adsorption properties of as received and ball-milled MoS_2_ nanopowders were examined through the adsorption of MB dye and phenol in aqueous solutions. In a typical experiment, 1.4 mg of MoS_2_ nanopowders were added in 2 mL of MB solution with a starting concentration of 1.5 × 10^−5^ M (4.8 ppm), at room temperature and with a pH of ~7.5. The test was run in parallel for each type of MoS_2_ nanopowders. At regular time intervals, the solutions were collected and centrifuged at 13000 rpm for 10 min, so to separate the nanopowders. In order to evaluate the adsorption of MB onto MoS_2_, the variation in the dye concentration was evaluated spectrophotometrically (using a PerkinElmer Lambda 45 UV-vis spectrophotometer) via the solution absorbance at 664 nm in the Lambert-Beer regime^54^. The dye adsorption on the beaker walls was also checked, as a reference, in the absence of the nanopowders. Every 20 min, the solutions were replaced with fresh MB solutions and the adsorption cycle was repeated with the same procedure. After three cycles, the MoS_2_ nanopowders were washed with NaOH 0.1 M for a few minutes, in order to restore the adsorption properties of the MoS_2_.

The aptitude of the investigated MoS_2_ nanopowders to adsorb phenol in aqueous solution was also tested, by measuring (using a Hach DR 3900 spectrophotometer) the residual concentration of phenol solution after 20 min of staying in contact with the nanopowders. 1.4 mg of MoS_2_ nanopowders were added in 2 mL of phenol solution (starting concentration of phenol: 1.5 × 10^−5^ M) at room temperature. Also in this case, before to perform the measure the powders were separated from the solutions by centrifuging them at 1300 rpm for 10 min. A blank experiment was done in parallel as control.

### Artemia salina acute toxicity test

Commercially avalaible *Artemia salina* dehydrated cysts were purchased from local aquarium store (ArtemiaCyst Blue Line, Italy). Cysts were first hydrated in ASPM seawater solution (ASPM is a synthetic seawater made of: NaCl = 26.4 g, KCl = 0.84 g, CaCl_2_·H_2_O = 1.67 g, MgCl·H_2_O = 4.6 g, MgSO_4_·7H_2_O = 5.58 g, NaHCO_3_ = 0.17 g and H_3_BO_3_ = 0.03 g) and then washed to separate the floating cysts (i.e., dead) from those that sink (i.e. alive). The sinking cysts were collected, and approximately 1 g of the precleaned cysts were incubated in 800 mL of ASPM solution seawater in a conical plastic container with graduations. A 1.500 lux daylight was provided continuously by a fluorescent lamp. Aeration was maintained by a small line extending to the bottom of the hatching device from an aquarium air pump. Under conditions of incubation at room temperature (26 ± 1 °C), gentle aeration and continuous illuminations, the nauplii hatched within 24 h. Two stock solutions of as received MoS_2_ (5 mg/mL) and ball-milled MoS_2_ 40 h (5 mg/mL) after dilution in ASPM solution, were prepared. Then, fresh suspensions with different concentrations of nanopowders (10^−1^ and 10^−2^ mg/mL) were made starting from the stock suspensions. These solutions were vortexed for 30 seconds, and then sonicated in an ultrasonic bath for about 20 minutes. 200 μL of each different concentrations of nanopowders solutions were added to the 96-well microplates. After that, 1 nauplius per well was added and incubated at 26 °C for 24/48 hours. The number of surviving nauplii in each well was counted under a stereomicroscope after 24/48 hours. A control group was also setup with ASPM seawater solution only. Larvae were not fed during the bioassays. The endpoint (immobility, i.e. death) was assessed at the end of the test by a stereomicroscope (a Leica EZ4): a nauplium was considered to be immobile or dead, if it could not move its antennae after slight agitation of the water for 10 seconds. Larvae that were completely motionless were counted as dead, and the percentages of mortality compared to the control were calculated. The death % of the crustacean for each concentration was calculated as follow: (n. dead nauplii/n. total animal treated) • 100. The collected data were analyzed for significance by one-way ANOVA test.

## Results and Discussion

The ball-milling process was applied to commercial MoS_2_ nanopowders, for 20 or 40 hours, in order to evaluate the effect of the process on the adsorption properties of the MoS_2_.

**Figure 1:**
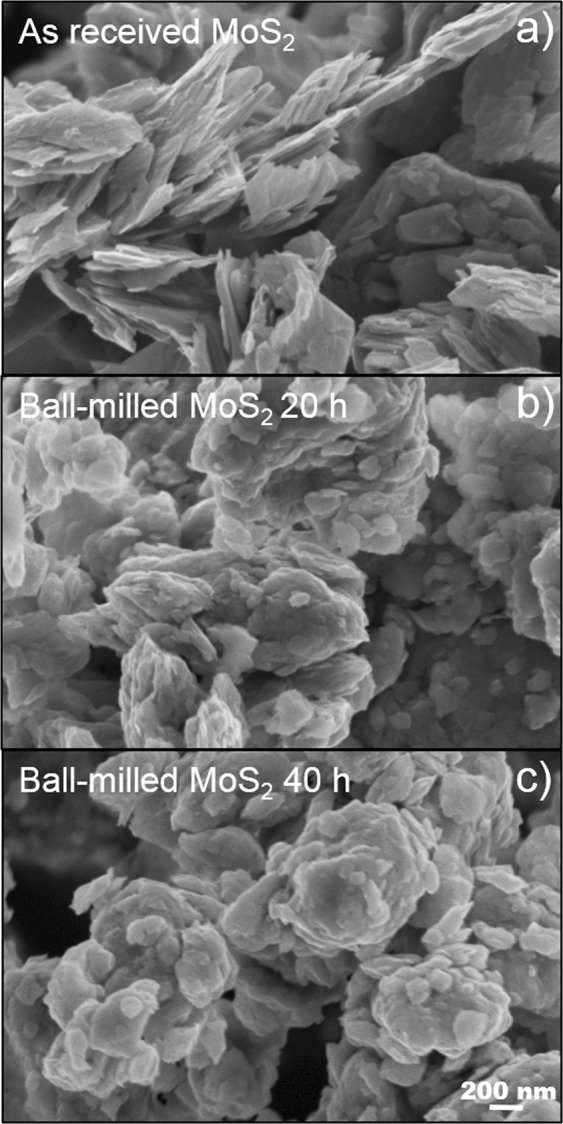
SEM images of as received MoS_2_ (**a**), ball-milled MoS_2_ for 20 hours (**b**), and ball-milled MoS_2_ for 40 hours.

SEM, in plan-view, was used to investigate the morphological alterations induced by the milling process. Figure 1a shows a SEM image of the MoS_2_ nanopowders before the ball-milling. Large MoS_2_ flakes (about 1 μm in size) together with little flakes (about 100 nm in size) can be seen. Figure 1b,c clearly evidence rounded flakes in comparison with the as received materials (Fig. 1a) as a result of the ball-milling process. Besides this evident difference between the as received and ball-milled nanopowders, SEM images do not show any other diversity, nor do reveal any variation in the size of the nanopowders, within the SEM resolution of course.

**Figure 2:**
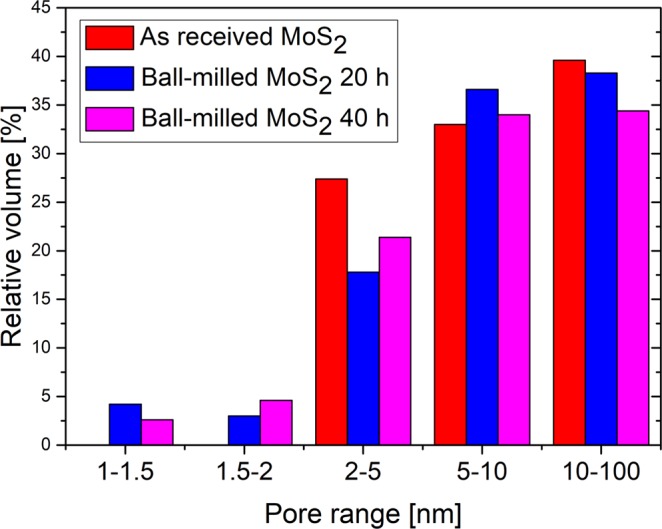
Pore size distribution for the as received MoS_2_ and the ball-milled MoS_2_ for 20 hours and 40 hours, respectively.

Consequently, in order to investigate an eventual reduction in size of the as-received MoS_2_ nanopowders thanks to the ball-milling process, the textural properties of the samples were measured through the BET and the Dollimore-Heal methods. In Table 1 the obtained specific surface area, pore specific volume and mean pore diameter values for the three different samples are reported. These results reveal an increase of the specific surface area after the ball-milling process. In particular, the increase of surface area is ~27% after 20 hours of ball-milling and ~42% after 40 hours of ball-milling compared to the obtained values for the as received nanopowders. As a consequence of the ball-milling process, a decrease of mean pores diameter and of pores volume was also detected if compared to the as received material, with the formation of micropores included in the 1–2 nm range for the 20 and 40 hours ball-milled materials, that are absent in the as received sample as reported in the Fig. 2.

**Table 1:** Specific surface area, pore specific volume and mean pore diameter values, of the as received MoS_2_ and the ball-milled MoS_2_ for 20 hours and 40 hours.

	Specific Surface Area	Pore Specific Volume	Mean Pore Diameter
As received MoS_2_	26 ± 1 m^2^/g	0.09 ± 0.02 cm^3^/g	11.2 ± 0.1 nm
Ball-milled MoS_2_ 20 h	33 ± 1 m^2^/g	0.06 ± 0.02 cm^3^/g	7.8 ± 0.1 nm
Ball-milled MoS_2_ 40 h	37 ± 1 m^2^/g	0.04 ± 0.02 cm^3^/g	6.9 ± 0.1 nm

**Figure 3:**
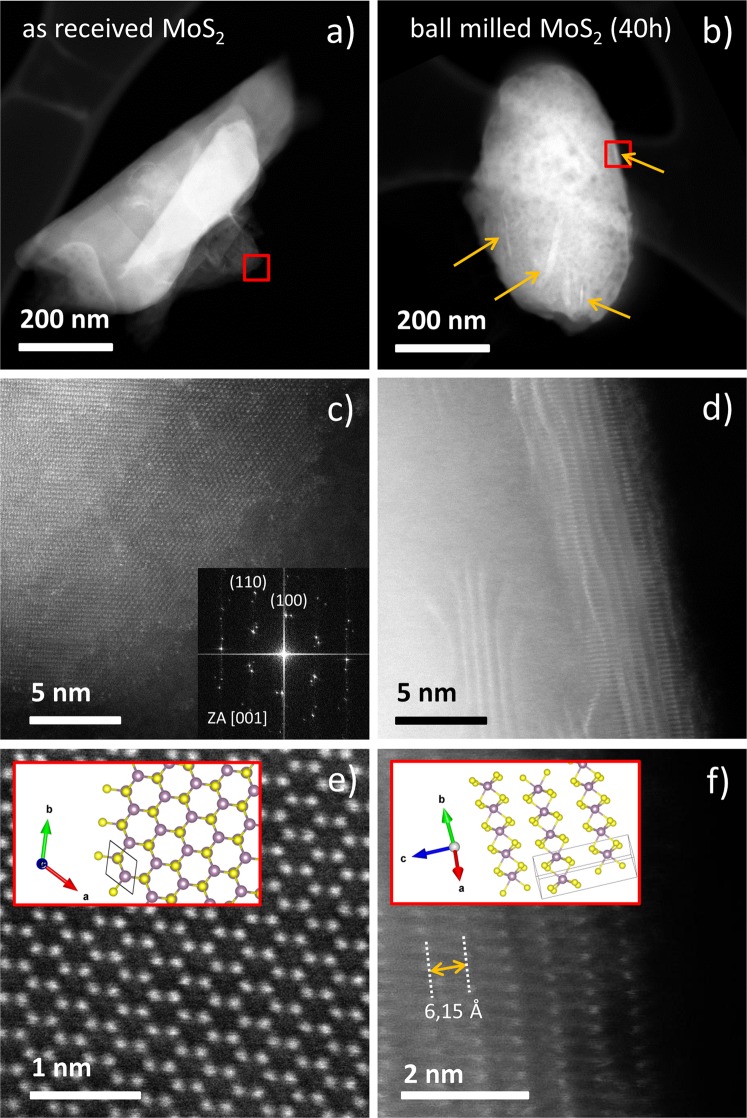
HAADF Z-contrast S/TEM images of as received MoS_2_ (**a-c-e**), and ball-milled for 40 hours (**b-d-f**). Low magnification of a single flaker of as received MoS2 (**a**); atomically resolved Z-contrast S/TEM image of the region indicated by a square in Figure 3a and his relative FFT (inset) (**c**); high magnification of the atomically resolved Z-contrast S/TEM image of Figure 3c with the simulated crystal structure (**e**). Low magnification of a single ball-milled flake (**b**); atomically resolved Z-contrast S/TEM image of the region indicated by a square in Figure 3b (**d**); high magnification of the atomically resolved Z-contrast S/TEM image of Figure 3d clearly showing the direct crystal-line structure (**f**).

In Fig. 3 we show the results of TEM analyses for the as received MoS_2_ flakes (a-c-e) and the ones exposed to 40 hours of ball-milling (b-d-f), respectively. The S/TEM Z-contrast images, obtained before the ball-milling process, show the MoS_2_ flakes having a flat shape (Fig. 3a), in particular the specimen looks to be formed of nano flakes with different area and shape stacked on top of each other. They assume a more rounded shape after the ball-milling exposure (Fig. 3b), and appear to be very crumpled, where randomly two different type of structures could be recognized. A first type of structure is reported to be as amorphous and porous-like. This kind of structure is usually present on a contaminated specimen by hydrocarbon components, which usually experiences the formation of voids, due to his sublimation under electron beam irradiation by TEM^55^. A second type of structures shows a number of random oriented bright stripes (arrows in Fig. 3b). In this case MoS_2_ lattice planes are compacted and randomly oriented due to ball-milling exposition, and differently by the as received specimen, where the lattice planes perfectly stacked and easy to be aligned with the electron beam of the TEM, here only the lattice planes accidentally aligned with the electron beam show a brighter contrast, while the others give an uniform contrast as background.

The high-resolution image in Fig. 3c and his relative fast Fourier transform (FFT), taken on a portion of the as received specimen, (see red square in Fig. 3a), shows the regular crystalline nature of the MoS_2_. In particular, the FFT shows two pattern corresponding to the P63/mmc space group^54^ aligned on the (001) zone axis. This means that two coplanar flakes are rotated by few degrees one other, around the a-axis. In Fig. 3e, HAADF Z-contrast STEM at higher magnification shows the direct atomic structure of the MoS_2_ crystal lattice that is the same as the one predicted by the theoretical reconstruction reported into the inset^56^. The absence of contrast modulation on the Z-contrast image in correspondence of the heavy Mo, compared with the light S atoms, implies the presence of AB stacking of MoS_2_ lattice planes, i.e. atoms of Mo and S are regularly alternated each other, along the same atomic column.

The presence of a regular atomic structure, as reported on the high-resolution S/TEM image of Fig. 3d, taken at a portion of the ball-milled flake evidenced by the red square in Fig. 3b, confirms the persistence of his crystalline nature. The in-plane view at this region permits to put in evidence the MoS_2_ layers stacked up along the c-axis. Due to the weak van der Waals forces, these layers, after the milling exposure, change their original distance between planes that is then not constantly maintained. In Fig. 3f a comparison between the real crystal structure from a non-damaged area and his theoretically reconstructed crystal structure^56^ is illustrated. The measured inter-planar distance of 6.15 Å is in good agreement with the one reported in the literature for MoS_2_^57^.

Summarizing the results of the TEM analysis, the ball-milling process modifies the overall structure order of the flakes. They go from a perfectly flat stacked crystalline structure to a round shaped one made by crumpled but still crystalline planes of MoS_2_. The thickness of the MoS_2_ flakes has been estimated by TEM analyses and resulted to be 5–6 nm.

**Figure 4:**
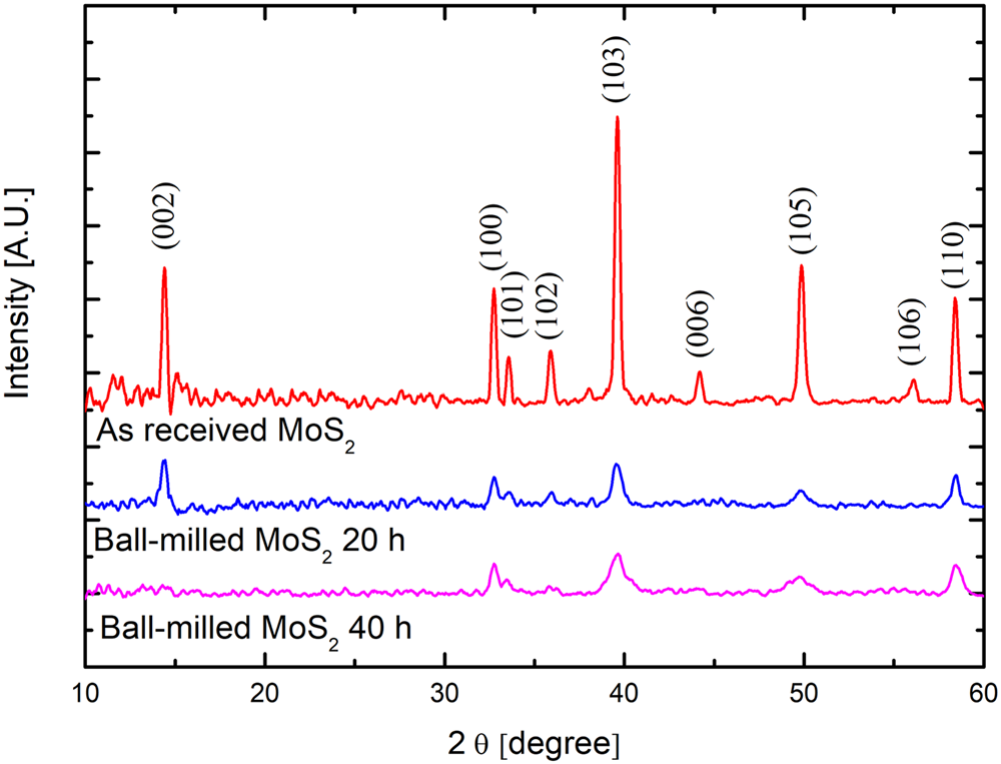
XRD pattern for as received MoS_2_, ball-milled MoS_2_ for 20 hours, and ball-milled MoS_2_ for 40 hours.

Figure 4 shows the XRD patterns of the as received MoS_2_ nanopowders (red line) and of the samples ball-milled for 20 hours (blue line) or 40 hours (magenta line). The spectrum of the as received sample shows all the reflections of molybdenite, as reported in JCPDS cards no. 37–1492: the (100), (103) and (110) reflections could be readily indexed as hexagonal MoS_2_^58,59^. Even if the crystallinity is maintained after the aggressive process of ball-milling, a decrease of all the peaks with the increase of the ball-milling time, is evident. In particular, the peak at 14.4° related to the (002) reflection^57^, associated with the sheet of MoS_2_, has a maximum intensity in the as received sample but gradually disappears with the ball-milling process, indicating that the MoS_2_ layers are not yet orderly stacked but splitted apart.

**Figure 5:**
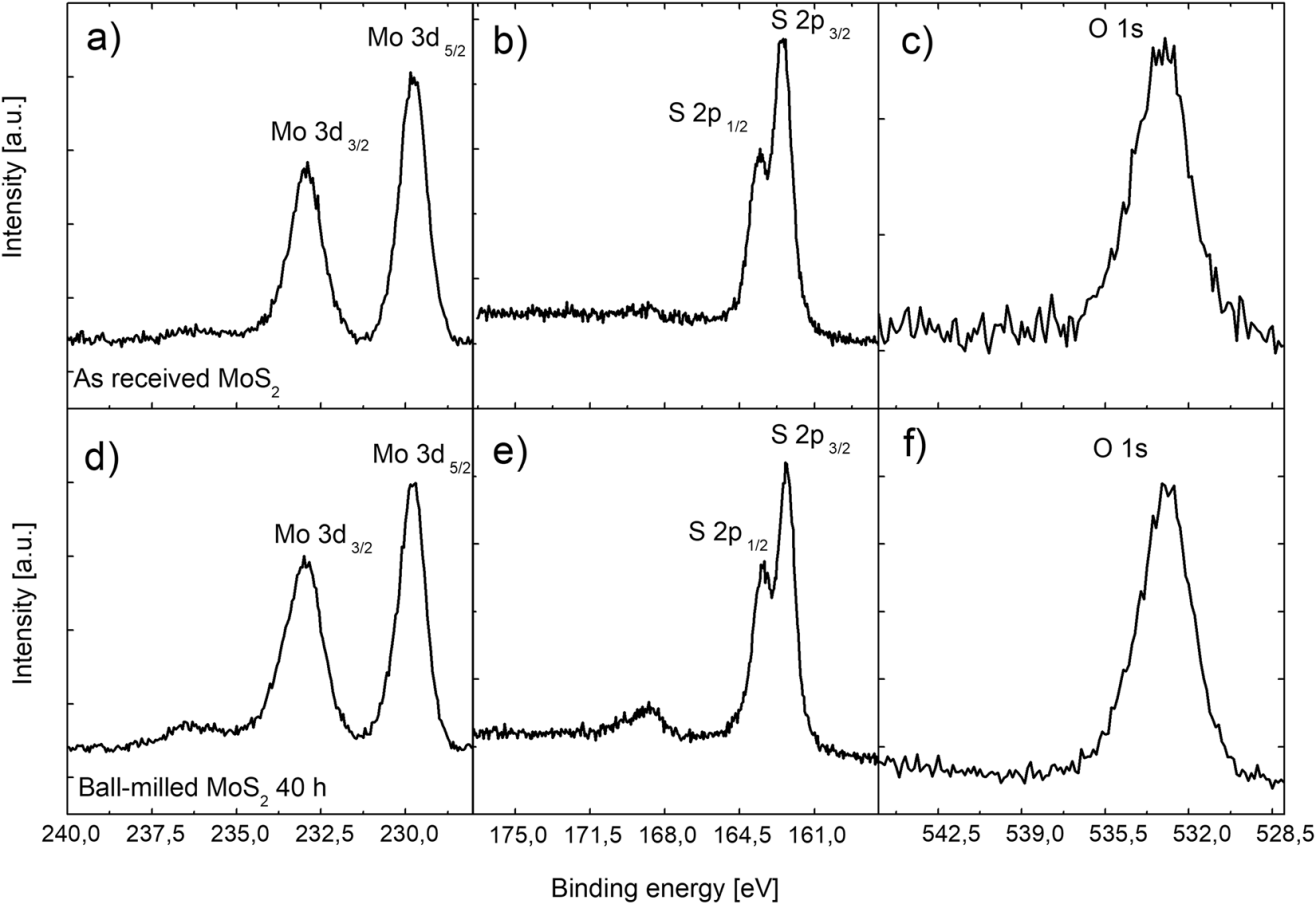
Mo 3d (**a**), S 2p (**b**) and O1s (**c**) spectra for the as received MoS_2_; Mo 3d (**d**), S 2p (**e**) and O1s (**f**) spectra for the ball-milled MoS_2_ for 40 hours.

XPS measurements were performed before and after the ball-milling for 40 hours, in order to investigate the surface chemical composition of the nanopowders. From the survey spectra of both samples (not reported here) the signals of O, C, Mo, and S were detected, so demonstrating that no contaminant is present neither in as received nanopowders nor in ball-milled powders (within the XPS resolution). In the well-resolved Mo 3d spectrum (Fig. 5a) of the as received sample, two peaks at 229.3 eV (Mo 3d_5/2_) and 232.4 eV (Mo 3d_3/2_) were observed and attributed to the Mo^4+^ oxidation state of MoS_2_. In the S 2p spectrum for the same sample (Fig. 5b), the peaks at 162.0 eV and 163.5 eV, attributed to S 2p_3/2_ and S 2p_1/2_ respectively, are associated with the divalent S in the MoS_2_. The same characteristic peaks were observed also for the samples submitted to ball-milling (Fig. 5d,e); the only difference is the presence of a bump in both Mo and S spectrum (peaked at ~236 eV and ~169 eV, respectively). This bump is probably due to a surface oxidation. The amount of oxidation was calculated by comparing the area of the peaks of oxidized species at 236 eV and at 169 eV with the totally area of Mo and S peaks, respectively. The results of this exercise gave an increase of 20% of oxidation in both regions. This result can be ascribed to the higher exposed surface area of the ball-milled material (as demonstrated by BET measurements). In order to investigate the eventual presence of new oxidative species induced by the ball-milling process, the oxygen regions are reported for both samples in Fig. 5c,f; this analysis does not show any other species.

**Figure 6:**
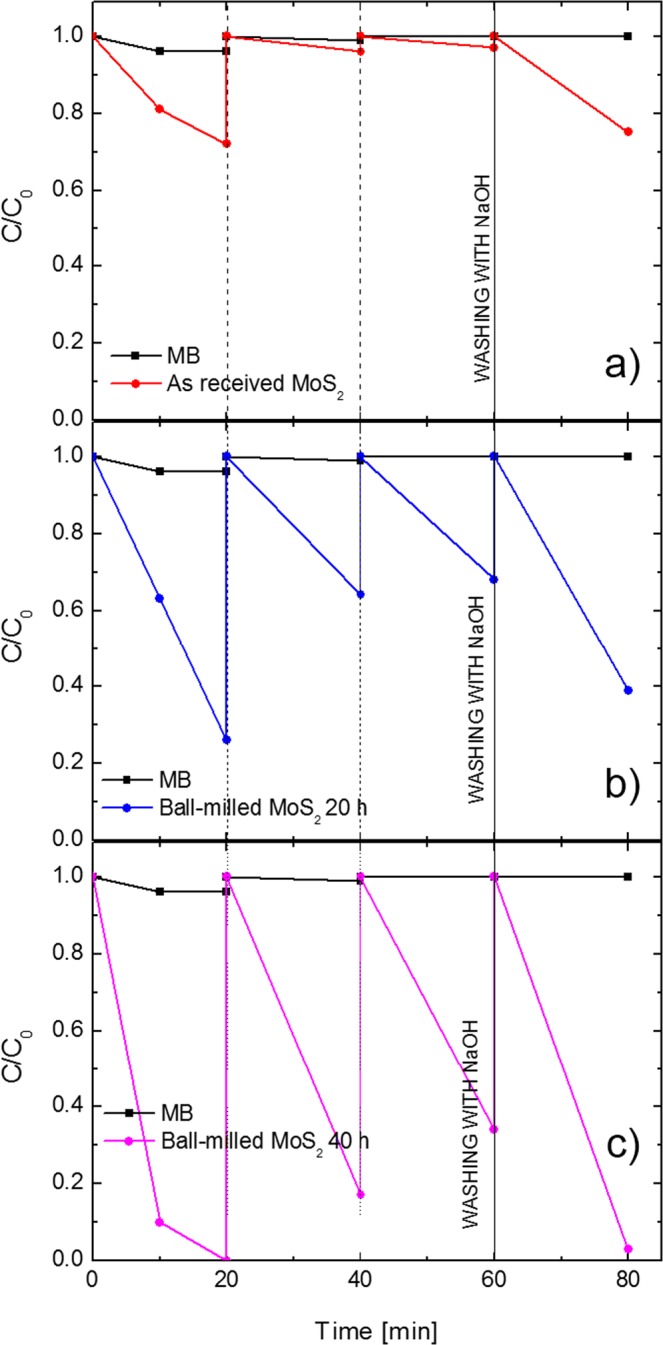
MB adsorption onto the as received MoS_2_ (**a**), ball-milled MoS_2_ for 20 hours (**b**), and ball-milled MoS_2_ for 40 hours (**c**). Vertical dashed lines indicate the change of the MB solution. Vertical solid line indicates the nanopowders washing step. The oblique lines are guide for eyes.

The adsorbance capacity of the investigated MoS_2_ nanopowders was tested through the adsorption of MB dye in aqueous solution. The obtained results are presented in Fig. 6, where the variation in the MB concentration as a function of the time is reported. The test was run in parallel for the as received MoS_2_ nanopowders (Fig. 6a), ball-milled MoS_2_ for 20 hours (Fig. 6b), and ball-milled MoS_2_ for 40 hours (Fig. 6c). In order to exclude any possible effects due to the adsorption of the organic dye on the beaker walls, the variation in the MB concentration in the absence of any adsorbent was also checked as reference and plotted as a black line in each graph. The color of the blue dye starts to fade in the presence of MoS_2_, thus suggesting the removal of the dye from water by its adsorption onto the surface of the nanopowders. In the first cycle, the solutions were collected after 10 min, centrifuged and measured using a spectrophotometer. The procedure was repeated after other 10 min. The adsorption of MB on the as received MoS_2_ was ~30% after 20 min (Fig. 6a). In the same time frame, ball-milled powders for 20 hours were able to adsorb ~80% of the dye (Fig. 6b). More interestingly, MB adsorption on ball-milled MoS_2_ nanopowders for 40 hours was found to be very fast and 100% of the dye was removed from water within only 20 min (Fig. 6c). Every 20 min the solutions were replaced with fresh MB solutions (this change is marked in the graphs with vertical dashed lines), and the adsorption cycles were repeated with the same procedure reported above. During the second cycle, the as received MoS_2_ removed less than 5% of MB, ball-milled MoS_2_ for 20 hours and 40 hours removed ~40% and ~80% of the dye, respectively. During the third cycle, the as received MoS_2_ continued to adsorb less than 5% of MB, ball-milled samples for 20 hours and 40 hours removed almost 40% and 70% of the dye, respectively. Since the adsorption process is a surface-driven process, the reduction of the adsorption capacity for all the samples is surely due to a reduction of the available sites for the physisorption process on the surface of the samples. With the main aim of reactivating the surface of the samples, and verify their reusability, the MB adsorbed on the nanopowders was removed by washing them for a few minutes with an aqueous solution of sodium hydroxide (the washing is marked with a vertical solid line in Fig. 6). We found that all tested samples recovered their starting adsorption performances, so demonstrating that the MoS_2_ could be easily recycled and consequently reused without any degradation of the adsorption sites. The little differences in the adsorption efficiency, if compared with the efficiency at the beginning of the test (for example, for the ball-milled MoS_2_ nanopowders for 40 hours the adsorbance efficiency changes from 100% to 97%), is due to a small loss of nanopowders during the centrifugation steps.

**Figure 7:**
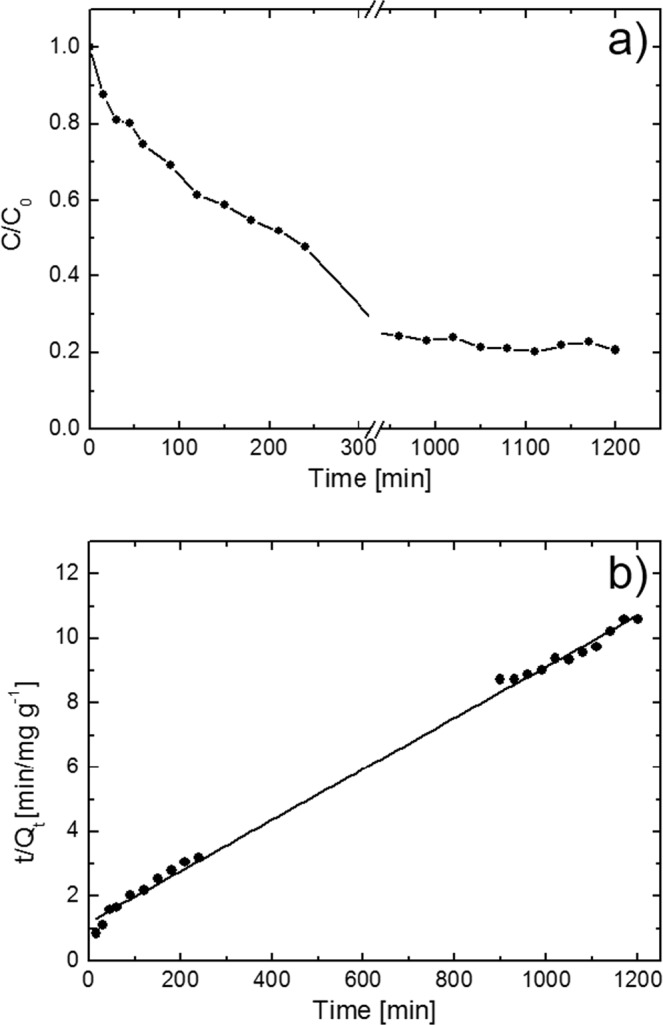
MB adsorption onto the ball-milled MoS_2_ for 40 hours (**a**) (the line is guide for eye); adsorption kinetic of MB onto the ball-milled MoS_2_ for 40 hours compared with the pseudo-second-order model (**b**).

We deeply investigated the adsorption kinetic and the adsorption capacity at the equilibrium of the ball-milled nanopowders. Adsorption tests were performed using 10 mL of aqueous solution with a MB concentration of 100 ppm. 7 mg of ball-milled MoS_2_ for 40 hours was used as adsorbent and added to the MB solution. Under these experimental conditions, the system is able to reach the equilibrium point, i.e. the point in which the dye adsorbed is equal to the dye desorbed by the adsorbent, so that the concentration of the dye in the solution remains constant. At regular time intervals, aliquots of the dispersion were collected and after dilution, the concentration of MB dye was examined spectrophotometrically until reaching the equilibrium point. The obtained results are reported in Fig. 7a. The adsorption capacity at the equilibrium, Q_e_ (mg g^−1^), was calculated by using the following equation:

1 $${Q}_{e}=({C}_{0}-{C}_{e})\times V/W$$

where, C_0_ (mg L^−1^) is the initial concentration of MB, C_e_ (mg L^−1^) is the concentration of MB at the equilibrium, V (L) is the volume of wastewater, W (g) is the amount of MoS_2_ used as adsorbent. At the equilibrium, the ball-milled MoS_2_ for 40 hours nanopowders have an adsorption capacity of ~113 mg g^−1^. This value is significantly higher of the adsorption capacity for many examples of MB adsorbent described in the literature^60–63^.

In the present study, linear pseudo-first-order (eq. 2), linear pseudo-second-order (eq. 3), and intraparticle diffusion (eq. 4) adsorption models were used to fit our experimental data. The models are expressed, respectively, by the following equations^64–66^:

2 $$\mathrm{ln}({Q}_{e}-{Q}_{t})=ln{Q}_{e}-{k}_{1}t$$

where Q_t_ (mg g^−1^) is the amount of dye adsorbed at time t (min), k_1_ is the pseudo-first-order rate constant (min^−1^).

3 $$\frac{t}{{Q}_{t}}=\frac{1}{{k}_{2}{Q}_{e}^{2}}+\frac{t}{{Q}_{e}}$$

where k_2_ is the pseudo-second-order rate constant (g mg^−1^ min^−1^).

4 $${Q}_{t}={k}_{id}{t}^{0.5}+C$$

where k_id_ is the intraparticle diffusion rate constant (mg g^−1^ min^−1/2^).

Based on these three models, curve fitting were performed. We found that our experimental data fit more closely to the pseudo-second-order model with R^2^ of 0.997 (Fig. 7b). Instead, for the pseudo-first-order and intraparticle diffusion models the R^2^ values are 0.834 and 0.967, respectively, revealing that these two models are not suitable to describe our adsorption process.

**Figure 8:**
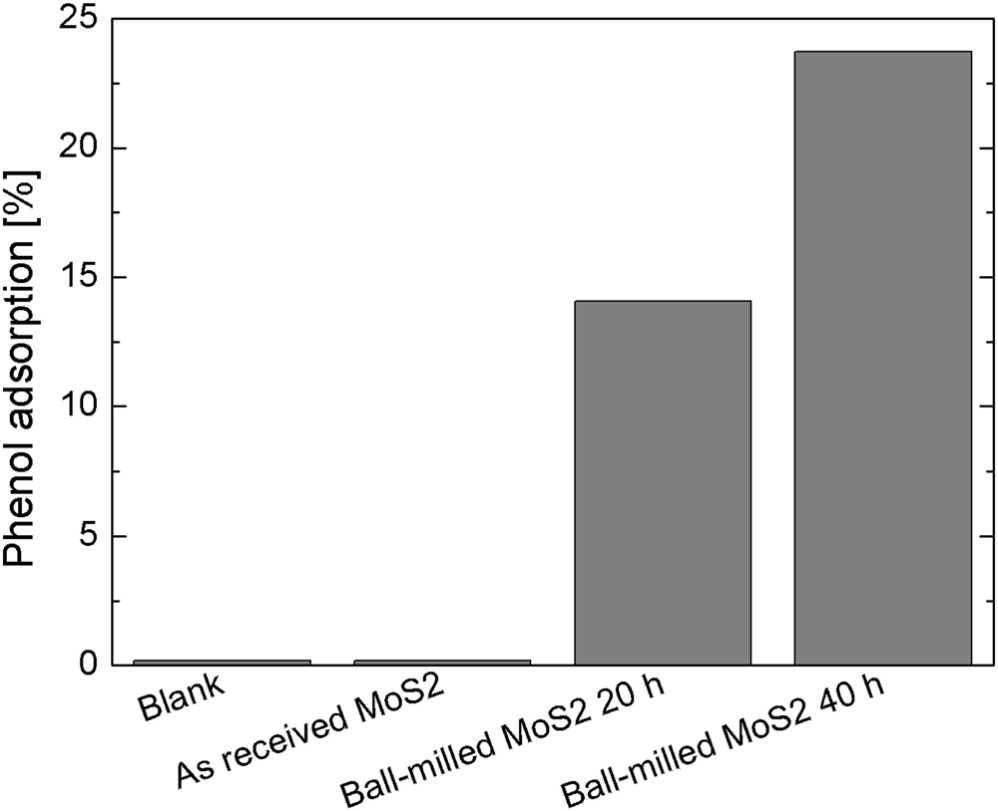
Phenol adsorption onto as received MoS_2_, ball-milled MoS_2_ for 20 or 40 hours.

The extremely high adsorption of ball-milled MoS_2_ was further tested for the adsorption of phenol, a toxic and water refractory pollutant, widely used in many industrial processes but hardly removed through common wastewater treatment methods^67^. After 20 min the solution was collected and centrifuged, while the residual phenol concentration was measured using a spectrophotometer. Figure 8 reports the percentage of adsorbed phenol by each sample, compared to a blank experiment, i.e. a phenol solution without nanopowders. The data clearly illustrates that the as received MoS_2_ nanopowders are not able to remove phenol at all. On the contrary, MoS_2_ nanopowders submitted to ball milling process for 20 and 40 hours are able to adsorb ~15% and ~25% of phenol, respectively, in only 20 min.

**Figure 9:**
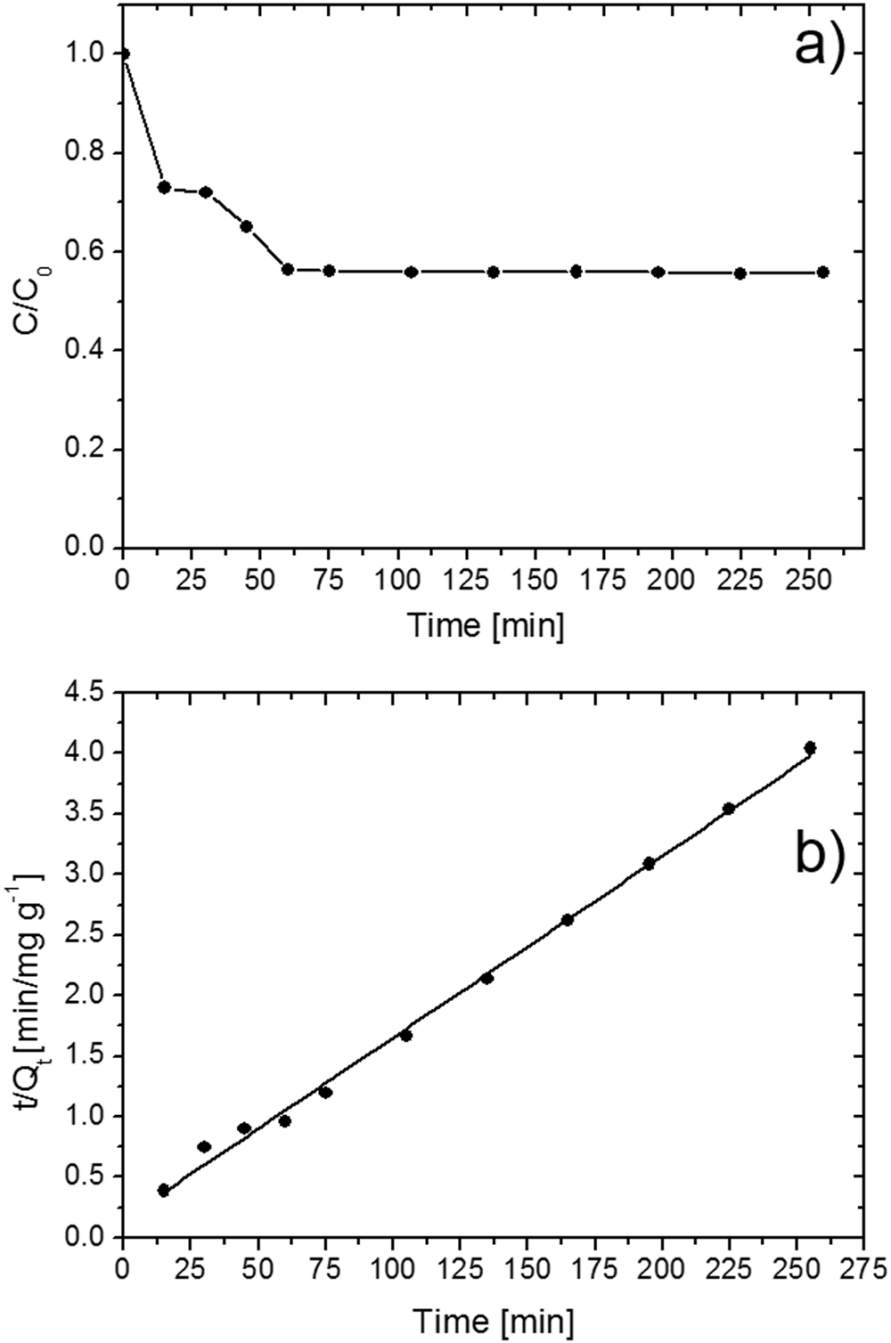
Phenol adsorption onto the ball-milled MoS_2_ for 40 hours (**a**) (the line is guide for eye); adsorption kinetic of phenol onto the ball-milled MoS_2_ for 40 hours compared with the pseudo-second-order model (**b**).

As it was done for MB adsorption, the adsorption kinetic and the adsorption capacity at the equilibrium for phenol was deeply investigated. The experiment was performed using 10 mL of aqueous solution of phenol 100 ppm in which 7 mg of ball-milled MoS_2_ for 40 hours were added. At regular time intervals, aliquots of the dispersion were collected and after dilution, the concentration of phenol was examined spectrophotometrically until reaching the equilibrium point. The obtained results are reported in the Fig. 9a. The adsorption capacity at the equilibrium was calculated by using eq. 1 and it resulted 63 mg/g, confirming the lower affinity of MoS_2_ towards phenol. Also in this case the experimental data fit more closely to the pseudo-second-order model (see Fig. 9b) with R^2^ of 0.996.

**Figure 10:**
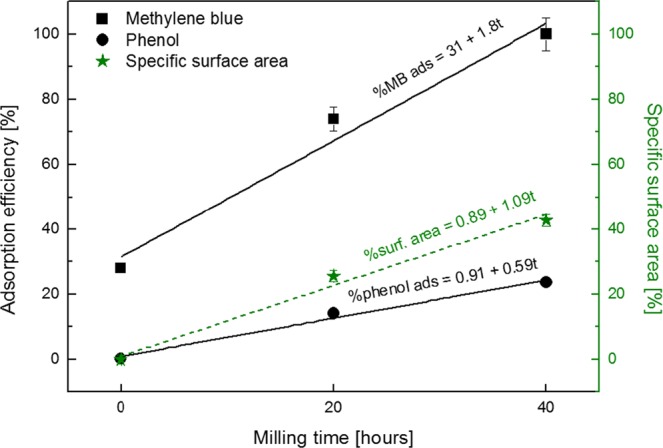
Efficiency of MB (black squares) and phenol (black circles) adsorption, and specific surface area increment of the samples (green stars) plotted as a function of the milling time. Black solid lines are the linear fits for MB and phenol adsorption efficiency, respectively. Green dashed line is the linear fit for the specific surface area increment. For each linear fit, the corresponding equation is indicated.

Figure 10 shows on the left vertical axis the % removal efficiency for MB dye and phenol as a function of the milling time. A linear trend can be easily detected for both contaminants; in particular the experimental data for MB can be fitted by the following straight line: %MB ads = 31 + 1.8 t, while the data for phenol can be fitted by the following straight line: %phenol ads = 0.91 + 0.59 t, where t is here the milling time. A higher efficiency in the adsorbance of MB compared to phenol can be clearly evidenced already for the as received samples. The lower efficiency in adsorbing phenol with respect to MB dye can be explained with an electrostatic repulsion between the negative charge density typical of the aromatic ring of the phenol and negative charge density on the sulfur atoms present in the MoS_2_ surface. On the contrary, the adsorption of MB is favorite thanks to the positive charge present in the MB molecule. Figure 10 also reports on the right vertical axis the specific surface area increment as a function of the milling time as obtained by BET measurements. The data can be fitted by the following straight line: %surf. area = 0.89 + 1.09 t. The observed correlation between the adsorption trend and the specific surface area trend clearly indicates that the adsorption efficiency strongly depends on the exposed surface area of the nanopowders. Higher exposed surface area means higher number of adsorption sites, and thus higher efficiency in the adsorbance of water contaminants. The error in the evaluation of both adsorption efficiency and specific surface area increment is within the symbol where it does not appear in the graph.

**Figure 11:**
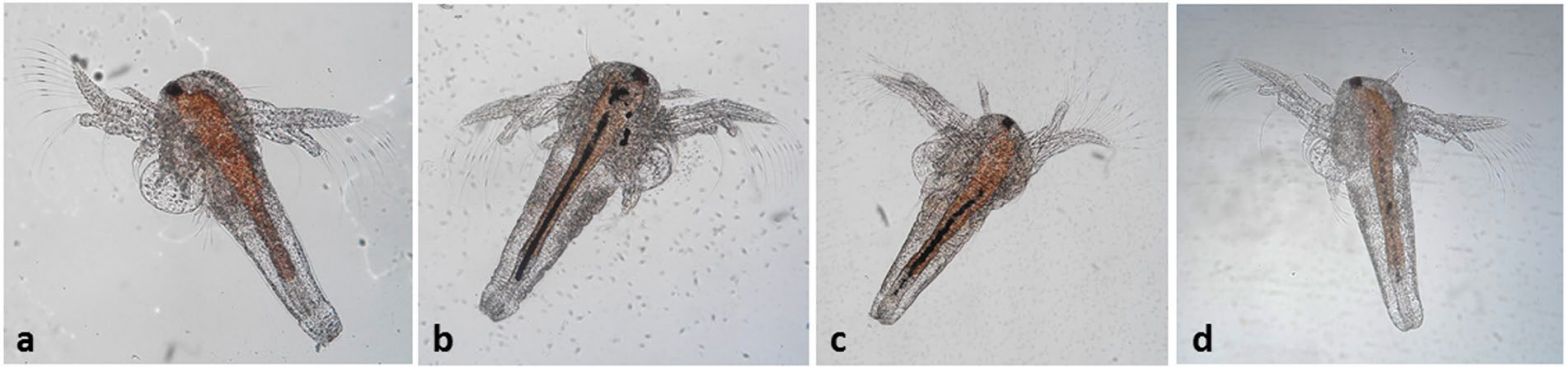
Artemia salina larvae: control at 24 hours (**a**), larve exposed to as received MoS_2_ at 24 hours (**b**), larve exposed to ball-milled MoS_2_ for 40 hours at 24 hours (**c**); larve exposed to ball-milled MoS_2_ nanopowders at 48 hours, in which is evident the reduction of the gut contents (**d**).

Finally, the potential toxicity of the as received and ball-milled MoS_2_ nanopowders was originally evaluated by testing the effects of the materials on microcrustacean *Artemia salina* in aquatic environment. *Artemia* is a non-selective filter-feeder organism that can readily ingest fine particles smaller than 50 μm^68^. For this reason, it is a model organism in toxicity assessment of nanoparticles. The accumulation of the nanomaterials into the *Artemia salina* was evaluated qualitatively at the end of the exposure (24 and 48 hours) thanks to a stereomicroscope equipped with a digital camera. After 24 hours, compared with the controls (Fig. 11a), the guts of the exposed larve to MoS_2_ nanopowders were filled with particles (Fig. 11b,c). The ingested particles appeared as a long strip of particles suggesting that even larger aggregates formed inside the guts. This effect was thought to be due to the reduced surface area as the powders agglomerated to microscale particles in solution and inside the guts. After 48 hours of exposure the nauplii eliminated the MoS_2_ nanopowders as displayed by Fig. 11d.

No mortality was found neither in control group nor in the exposed organisms. The percentages of immobilized nauplii are reported in Table 2. The death % is not significant for both tested nanopowders.

**Table 2:** Percentages of immobilized nauplii after exposition to as received and ball milled MoS_2_ at two different concentrations for both 24 and 48 hours.

	10^−1^		10^−2^	
	24 h	48 h	24 h	48 h
As received MoS_2_	1%	0%	0%	0%
Ball milled MoS_2_ 40 h	1%	0%	0%	3%

## Conclusions

Commercial MoS_2_ nanopowders were processed by ball-milling for 20 or 40 hours. The milling process induced a significantly enhancement of the adsorption properties of MoS_2_ nanopowders. In particular, in only 20 minutes the MoS_2_ ball-milled for 40 hours was able to remove by adsorption 100% of MB dye initially presents in the aqueous solution. In addition, the adsorption capacity of MoS_2_ towards phenol was steeply intensified by ball-milling. Thanks to an accurate characterization of the investigated materials, the improved performance of the ball-milled nanopowders was correlated to the increase of the specific surface area, induced by the ball-milling process. Importantly, the recyclability tests revealed that the MoS_2_ nanopowders can be easily regenerated and reused without losing the adsorption capacity. The non-toxicity of the studied nanopowders was established by testing the effect of the material on microcrustacean *Artemia salina* in aquatic environment. The present original results demonstrate that the highly-scalable dry ball-milling process can be used for the preparation of highly-efficient adsorbents. This material can be fruitfully applied in wastewater treatment.
